# “The understanding and attitude toward epilepsy in the Souss‐Massa region of Morocco”

**DOI:** 10.1002/epi4.12709

**Published:** 2023-02-27

**Authors:** Amina Tani, Nawal Adali

**Affiliations:** ^1^ Laboratory of Health Sciences Research Ibn Zohr University Agadir Morocco; ^2^ Faculty of Medicine and Pharmacy of Agadir Ibn Zohr University Agadir Morocco

**Keywords:** attitude, epilepsy, knowledge, myths

## Abstract

**Objective:**

Sociocultural beliefs about epilepsy impede medical treatment and social acceptance of people with epilepsy. These barriers can lead to disability and contribute to the stigmatization of people with epilepsy.

Assessing knowledge, myths, and attitude about epilepsy and associated factors among adults in Souss‐Massa, Morocco.

**Methods:**

We conducted a cross‐sectional study in which data were collected from a sample of 385 individuals in the Souss‐Massa region of Southern Morocco. Data were collected through face‐to‐face interviews using a structured questionnaire. The analysis of the collected questionnaires was processed using IBM SPSS Statistics 26.

**Results:**

385 responses were included in the analysis. The results reveal a lack of knowledge, an unfavorable attitude, and the persistence of myths and misunderstandings about epilepsy. The findings of this study suggest that good knowledge was significantly associated with sociodemographic factors: the age range of more than 54 years and health professional status. In addition, the favorable attitude toward epilepsy correlates with the sociodemographic factors: male gender and urban residential area.

**Significance:**

The main finding of this study indicates insufficient knowledge, the persistence of myth, and an unfavorable attitude about epilepsy. Therefore, the application of public awareness programs about epilepsy in urban and rural areas is necessary.

**Key Point**

- Sociocultural beliefs about epilepsy impede medical treatment and social acceptance of people with epilepsy.
- In this study, we investigated Moroccans' attitudes and understanding regarding epilepsy and the social determinants that may influence them.
- The results reveal insufficient knowledge, unfavorable attitude, and the persistence of myths and misunderstandings about epilepsy.
- Good knowledge was significantly associated with sociodemographic factors: age range of more than 54 years and health professional status.
- The favorable attitude toward epilepsy significantly correlates with the sociodemographic factors: male gender and urban residential area.

## 1 INTRODUCTION

Epilepsy is a brain disorder characterized by an enduring predisposition to generate epileptic seizures. Moreover, this disorder has neurobiological, cognitive, psychological, and social consequences.^1^ Epilepsy affects more than 70 million people worldwide, nearly 80% of those who live in low‐ and middle‐income countries. Besides, anyone can develop epilepsy. Since it affects both men and women, regardless of race, ethnicity, and age.^2^, ^3^, ^4^

Epilepsy is often related to the misconception, fear, stigma, and myths in the general population and even among healthcare professionals.^5^, ^6^, ^7^, ^8^ In many parts of the world, and primarily in low‐ and middle‐income countries, more than 75% of people with active epilepsy are untreated, which is a health and economic burden.^9^

Indeed, several psychosocial and cultural factors are important determinants of the clinical course of epilepsy and are often among the main barriers to appropriate care and treatment. Again, false beliefs about epilepsy affect the social acceptance of people with epilepsy. As a result, the social stigma attached to epilepsy can limit their contribution to society. Usually, people with epilepsy have problems with employment, education, and social relationships. These difficulties can lead to disability and low quality of life. However, several studies have found that misinformation about epilepsy is associated with stigma, which suggests that improving knowledge could have a positive impact.^10^

In addition, understanding the variation in cultural understandings of epilepsy may help provide effective management.^3^ However, in Morocco, studies on the perception of epilepsy are rare.

Therefore, this study was done to determine the knowledge, myths, misunderstandings, and attitudes toward epilepsy among the Moroccan people and how the respondent's sociodemographic characteristics influenced these results.

The findings of this study will provide a thorough understanding of the quantification of the false beliefs concerning epilepsy among Moroccan individuals, which is essential before the implementation of epilepsy education for the public.

## 2 MATERIALS AND METHODS

### 2.1 Study design

It was a descriptive cross‐sectional study conducted in the period between January 2021 and August 2021.

### 2.2 Study setting

The study was conducted in the Souss‐Massa region in southern Morocco. This region has an area of 17 820 km^2^ and includes six provinces. According to the last census in 2014, the legal population of this region was established with 2 676 847 inhabitants.

### 2.3 Sampling size and procedure

The sample size calculated was 385, for a confidence level of 95%, a Z‐score = 1.96, and a margin of error at 5% with a proportion (p) = 0.05. A sample of 405 was selected to adjust for possible incomplete responses using multistage stratified clustered sampling from the six provinces: Agadir Ida‐Outanane, Chtouka‐Ait Baha, Inzegane‐Ait Melloul, Taroudant, Tata, and Tiznit. The six provinces were not equally represented since the number of people in each was distinct. This involved randomly selecting houses, schools, universities, gardens, hospitals, and shopping centers in each province. Then, individuals were contacted and explained the purpose of the study. They felt assured about confidentiality and agreed to participate in the study. The data were collected via face‐to‐face structured interviews with the respondents.

### 2.4 Study subjects

People with an age range of fifteen years and older from the Souss‐Massa region, who were available during the data collection period, were study subjects. Individuals who were unwilling or unable to communicate were excluded from the study.

### 2.5 Data collection instruments

#### 2.5.1 Data collection tools and measurement

To measure knowledge and attitudes toward epilepsy, a set of questions was compiled from documented research, related articles, and background locally on epilepsy. The questionnaire was developed by authors, first in English, then it was translated into two local languages of the Souss‐Massa region (Arabic dialect and Berber). Both versions have been checked and revised by a group of experts: doctors, nurses, Arabic language teachers, and Berber language experts. The two versions were translated back into English to verify their accuracy and conformity. Then, the questionnaire was tested on 20 people mastering the two local languages (Moroccan dialect and Berber) but not included in the sample. This test allowed adjustments and rectifications to ensure linguistic comprehensibility and social acceptability.

This questionnaire includes first an introduction that explains the interest of the study. After the participant's agreement, they would answer 19 items divided into four parts. There are several open and closed questions. The first part concerns questions that target the sociodemographic characteristics of the participants. Such as gender, age in years, educational level, health, professional status, and Residential area. The second part concerns the experience of epilepsy (having epilepsy or knowing a person with epilepsy).

The third part targets the knowledge, myths, and practices related to epilepsy. In effect, this part includes yes/no questions and multiple‐choice questions that admit several correct modalities according to the current scientific knowledge on the definition of epilepsy, characteristics of epilepsy, causes of epilepsy, types of epilepsy, types of seizures, treatments for epilepsy, and what to do when faced with an epileptic person in crisis and the consequences of epilepsy. The fourth and last part contains three elements with questions; about participants' attitudes toward patients with epilepsy. The first element concerns the acceptance that epileptic patients can lead an ordinary social life (getting married, having children, etc.) The second element about how much could the patient with epilepsy embrace the fact that he can lead a normal professional life (to work, to be responsible, etc.) The third element is to admit that people with epilepsy can be considered as people with stable moods.

#### 2.5.2 How did we create the scales to measure the epilepsy level of knowledge and attitude scales

On the one hand, to measure the epilepsy level of knowledge, we assigned to each dimension of measurement a score of “one” point for the answer in agreement with the current scientific knowledge and “zero” points for the one that is not consistent with the current scientific knowledge. For multiple‐choice questions that admit several correct modalities, we assigned part of the score “one” point distributed in equity according to the number of valid modalities. Thus, a “one” point score is awarded when all correct modalities are selected. The composite score of knowledge range from 0 to 10 points. It was dichotomized with a score of ≥50% as good knowledge and a score of <50% as poor knowledge. Cronbach's alpha is a reliability coefficient presented by Cronbach.^11^ It allows for measuring the internal consistency of tests and measures.^11^ In our study, it was used to assess the consistency of the epilepsy level of knowledge, and we found 0.728.^12^, ^13^

On the other hand, to measure the attitudes scales toward epileptic patients, we have assigned to each measurement dimension a “one” point score for the attitude favorable attitude response and “zero” points for the unfavorable attitude response. The composite score of attitude toward epilepsy patients ranges from 0 to 3 points. It was classified with a score of ≥50% as a favorable attitude and a score of 50% as an unfavorable attitude. The internal consistency (reliability) of the attitude item was checked by using Cronbach alpha, and we found 0,858.^12^, ^13^

#### 2.5.3 Data analysis procedure

Data were coded and entered into the EPI info statistical software, then exported to the SPSS windows version 26 program for analysis. Descriptive and inferential statistics were used to represent the data. Descriptive statistics, such as frequency and percentage, were used to summarize the sociodemographic characteristics of the study participants. Binary and multivariable logistic regression were applied. Binary logistic regression and adjusted odds ratio with a 95% confidence interval was used to identify factors associated with epilepsy‐related myths and misunderstandings.

Multivariable logistic regressions were used to analyze the results of knowledge and attitudes about epilepsy to identify significant factors for the dependent variables. The dependent variables were attitude (favorable and unfavorable) and knowledge (good and bad). The independent variables were sociodemographic factors such as gender, age, education level, health professional status, and area of residence. *P* values less than 0.05 were considered statistically significant, with the strength of the association presented by the adjusted odds ratio.

## 3 RESULTS

Four hundred and six responses were received and reviewed for eligibility. Twenty‐one respondents were excluded from the analysis due to missing data, while the rest were included.

### 3.1 Sociodemographic distribution of respondents

**FIGURE 1:**
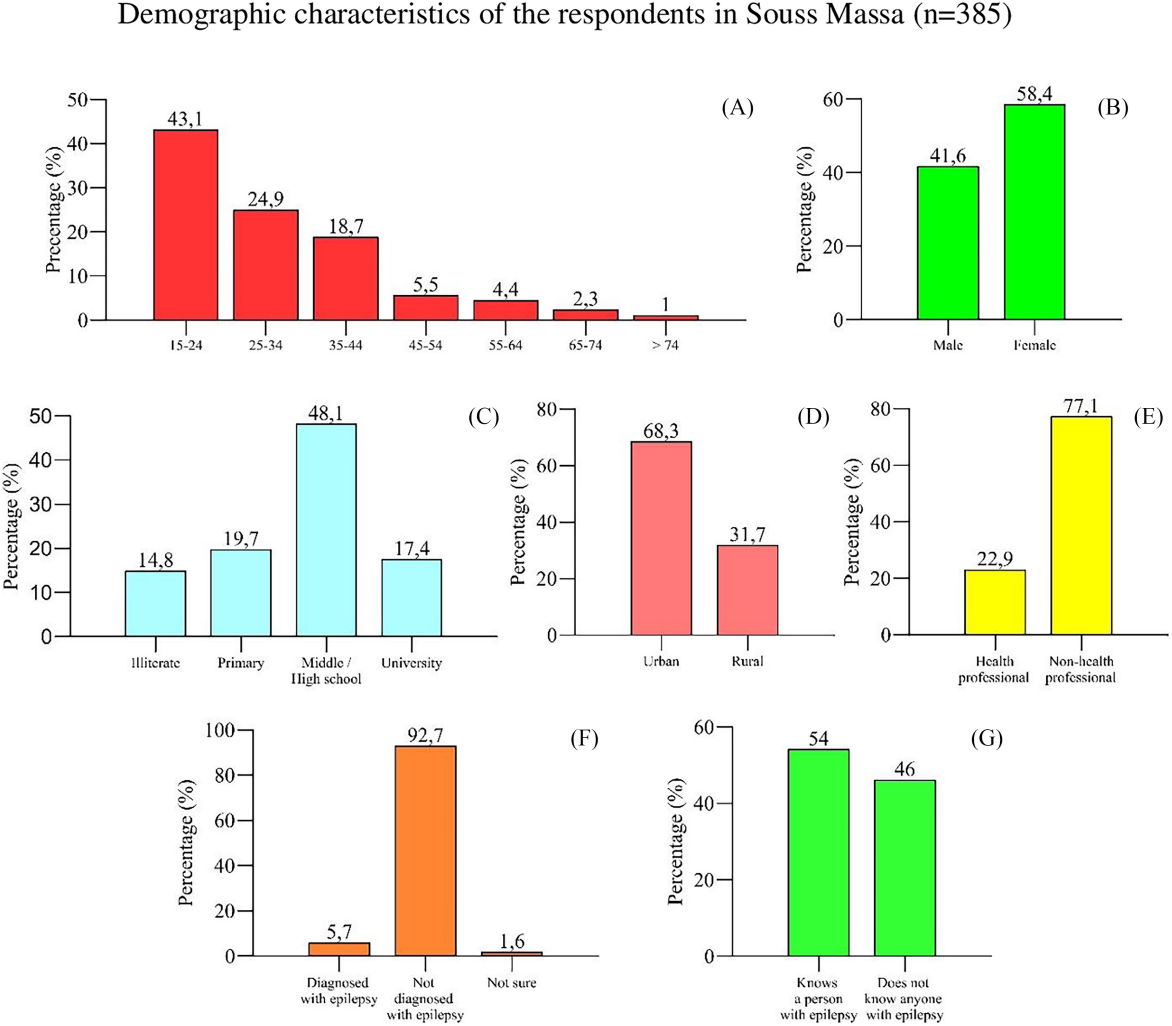
Demographic characteristics of the respondents in Souss‐Massa (n = 385). %: percent; A: age (years) of participants; B: gender of participants; C: participants’ educational level; D: participants’ origin; E: health professional status; F: participants with epilepsy; G: knowing a person with epilepsy.

According to Figure 1. Demographic characteristics of the respondents, 385 people participated in this study. About a third of the participants were of rural origin. The mean age of the sample was 30.76 years (range 15–80). More than half of the subjects were women (58.4%). About a quarter of the participants were health professionals. In addition, 22 people with epilepsy or a seizure disorder completed the survey, 5.7% of the sample, which is higher than estimates based on Morocco's prevalence of epilepsy alone. Again, 54% of participants knew people with epilepsy. In addition, the level of education was variable. About half of the participants (48.1%) had a university degree, compared to 14.8% who had no education, 17.4% who had primary education, and 19.7% who had a high school education.

### 3.2 The public's knowledge, myths and perception toward epilepsy

Through this study, about twenty names are used by the population of the region of Souss‐Massa, to designate epilepsy. However, these names in Berber, Moroccan dialect, and Arabic are related to the supernatural. Percentages of people responding correctly to each of the knowledge units are listed in Table 1.

**TABLE 1:** Public's knowledge, myths, and perceptions about epilepsy.

Characteristics	n	%	Characteristics	n	%
Definition of epilepsy			Can epilepsy be controlled with treatment?		
			Yes	240	62.3%
Neurological disease	272	58.5%	No	31	8.1%
Psychiatric disease	75	16.1%	I don’t know	114	29.6%
Madness	62	13.3%	How to treat epileptic patients?		
Retard mental	23	4.9%	Antiepileptic drugs	254	34.0%
I don’t know	33	7.1%	Surgery	75	10.0%
characteristics of epilepsy			Ketogenic diet	22	2.9%
			Traditional treatment	35	4.7%
Epilepsy is a dangerous disease	68	32.2%	Quranic therapy	131	17.5%
Epilepsy is a chronic disease	37	17.5%	Visit or stay in mausoleum	61	8.2%
Epilepsy is a common disease	32	15.2%	Visit the Faqih	81	10.8%
Epilepsy is a rare disease	32	15.2%	Other treatment	16	2.1%
I don’'t know	41	19.4%	I don’'t know	51	6.8%
The age at which epilepsy can appear			What can be done for seizure first aid?		
Epilepsy may occur in children	85	18.1%	Stay away from the person until the crisis is over	67	5.6%
Epilepsy may occur in adults	59	12.6%			
Epilepsy can occur in the elderly	23	4.9%	Trying to stop the seizure movements	51	4.3%
Epilepsy can occur at any age	243	51.7%			
I don’'t know	60	12.8%	Moving the person in the midst of a seizure	18	1.5%
Etiology of epilepsy					
Unknown	76	8.8%	Keeping dangerous objects Away	175	14.7%
Genetics	87	10.1%			
Head trauma	160	18.6%	Loosen light clothes around the neck	169	14.2%
Brain disease	171	19.9%			
Developmental disease	64	7.5%	Place a soft support under his head	93	7.8%
Possession (Jinn)	132	15.4%			
Bewitchment	108	12.6%	Putting honey, medicine, water, in the mouth of a person in crisis	75	6.3%
I don’t know	61	7.1%			
Able to recognize types of epilepsy			Putting a key in the hand of a person having a seizure	84	7.1%
Generalized	131	26.4%			
Focal	78	15.7%	Stay close to the person having the seizure, note the time of onset, and monitor the	164	13.8%
Generalized and focal	65	13.1%			
Unknown	27	5.4%			
I don’'t know	195	39.3%	Place the person in the lateral position as soon as possible	68	5.7%
Able to recognize types of seizures					
Emotional or sensory seizures	43	4.9%	If the seizure exceeds 5 minutes and the person in seizure has not regained consciousness, you must call for help	166	14.0%
Focal seizures	120	13.6%			
Absence seizures	66	7.5%			
Spasm seizures	129	14.6%			
Myoclonic seizures	155	17.6			
Tonic–clonic seizures	232	26.3%	I don’t know	59	5%

Abbreviations: %, percent; n, number.

Regarding the definition of epilepsy, 58.5% of respondents confirmed it is a neurological disorder, and 16.1% think it is more of a psychiatric disorder. While 13.3% assume that epilepsy is synonymous with “craziness.” Additionally, 39.3% of participants were unaware of the types of epilepsy and seizures.

Also, among the causes of epilepsy mentioned by the participants: were genetics at 10.1%, brain diseases at 19.9%, and head trauma at 18.6%. Lastly, bewitchment and possession (Jinn) were at 12.6% and 15.4%, respectively. As for the treatment, 34% of participants offered antiepileptic drugs while 10% indicated surgery, compared to 17.5% who offered spiritual therapies through the Quran. Concerning the management of an epileptic seizure, 13.8% of participants suggested staying close to the person having the crisis, noting the time of its onset, and monitoring it, while 7.1% suggested putting a key in the hand of the concerned person. Finally, 6.3% of participants suggested putting honey, water, or medicine in the person's mouth in the case of a crisis.

According to the results, the participants think that people with epilepsy have cognitive disorders, depression, and anxiety, 24.3%, 20.7%, and 29.4%, respectively.

### 3.3 Public attitude toward people with epilepsy

To learn more about the public's attitude regarding the impact of epilepsy on the social and professional lives of epileptic people and their moods, respondents answered several questions. Besides, only a quarter of the participants said that epileptic people who are undergoing proper treatment and medical care could lead an ordinary social and professional lives and have a stable mood.

### 3.4 Attitude, knowledge and myth toward epilepsy

#### 3.4.1 Result of the epilepsy level of knowledge and attitude scales

The epilepsy level of knowledge and attitude scales used in our study demonstrate that only 27.79% and 10.39% of respondents had good knowledge, and favorable attitudes to epilepsy, respectively, as shown in Tables 2 and 3.

**TABLE 2:** Answers to items about attitude toward people with epilepsy.

Questions	Yes (n) (%)
Do you admit that an epileptic person undergoing a medical treatment and a follow‐up can lead an ordinary social life (get married, have children, etc.)?	187 (29.9%)
Do you admit that an epileptic undergoing a medical treatment and a follow‐up can lead an ordinary professional life (work, study, be a leader, etc.)?	153 (24.5%)
Would you consider a person with epilepsy, who is undergoing a medical treatment as an ordinary person with a stable mood?	153 (24.5%)

Abbreviations: %, percent; n, number.

**TABLE 3:** Factors associated with good knowledge toward epilepsy.

Independent variable	Category	Dependent variable (knowledge)		OR (CI = 95%)	*P*‐value
		Bad	Good		
Gender	Male (%)	30.39	11.17	Reference	
	Female (%)	41.82	16.62	0.85	0.54
Age	15–34 (%)	50.91	17.40	Reference	0.02
	35–54 (%)	16.36	7.27	1.27	0.47
	More than 54 (%)	4.94	3.12	9.15	0.03
Educational level	Illiterate	12.21	2.60	Reference	0.14
	Primary	14.29	3.12	2.42	0.15
	Secondary/high school	17.14	2.60	2.48	0.24
	University	28.57	19.48	6.54	0.1
Residential area	Rural	25.45	6.23	Reference	
	Urban	46.75	21.56	1.315	0.866
Profession	Not a health professional	62.60	14.55	Reference	
	Health professional	9.61	13.25	4.07	0.001

*Note*: The significant result was at *P* ≤ 0.05.

Abbreviation: OR, odds ratio.

#### 3.4.2 Factors associated with a good knowledge of epilepsy

The results of the regression between knowledge of epilepsy and other factors are summarized in Table 2. Multivariate results were reasonably consistent with univariate results (subgroup analyses). Overall, good knowledge was significantly associated with sociodemographic factors: age range of more than 54 years (OR = 9.15 and *P*‐Value = 0.03) and Health professional status (OR = 4.07 and *P*‐Value <0.001). Regarding the gender of the participants in the study, our findings show that gender is a neutral factor (OR = 0.85 and *P*‐Value = 0.54). Also, our results show that the Residential urban area is a neutral factor (OR = 1.31 and *P*‐value = 0.86). Also, university educational level category is a neutral factor (OR = 6.54 and *P*‐value = 0.1). However, these findings may be subject to bias due to the small size of the sample.

#### 3.4.3 Factors associated with a favorable attitude toward epilepsy

The Multivariate analysis of the results in the study (Table 4) shows that favorable attitudes toward epilepsy were significantly associated with sociodemographic factors: male gender (OR = 0.5 and *P*‐Value = 0.007), Residential urban area (OR = 2.55 and *P*‐Value = 0.01). Regarding the age of the participants in the study, our findings show no statistical significance between the different age categories of participants and having favorable attitudes (*P* > 0.05). Thus, age is a neutral factor. Besides, no statistical significance is associated with Health professional status (*P* > 0.05), so it is a neutral factor. Similarly, for educational level category show no statistical significance (*P* > 0.05).

**TABLE 4:** Factors associated with a favorable attitude toward epilepsy.

Independent variable	Category	Dependent variable (attitude)		OR (CI = 95%)	*P*‐value
		Unfavorable	Favorable		
Gender	Male (%)	37.92	3.64	Reference	
	Female (%)	51.69	6.75	0.50	0.007
Age	15–34 (%)	61.30	7.01	Reference	0.28
	35–54 (%)	21.04	2.60	1.635	0.12
	More than 54 (%)	7.27	0.78	1.652	0.45
Educational level	Illiterate	14.29	0.52	Reference	0.72
	Primary	16.62	0.78	1.316	0.64
	Secondary/high school	19.22	0.52	1.517	0.52
	University	39.48	8.57	3.132	0.07
Residential area	Rural	30.39	1.30	Reference	
	Urban	59.22	9.09	2.557	0.01
Profession	Not a health professional	73.77	3.38	Reference	
	Health professional	15.84	7.01	2.208	0.1

*Note*: *Significant result at *P* ≤ 0.05.

Abbreviation: OR, odds ratio.

### 3.5 Factors associated with myths and misunderstandings toward epilepsy

Bivariate logistic regression was conducted to assess the association between the myth and misunderstandings about epilepsy and other sociodemographic variables. The analysis of the data from the present study showed a statistically significant association (*P* ≤ 0.05) between sociodemographic variables (gender male, non‐health professional status, Illiterate category, rural residential area) and the choice of propositions: “That epilepsy is synonymous with madness,” “That bewitchment and possession (Jinn) are among the causes of epilepsy,” “Those traditional and spiritual treatments are among the remedies,” “The recourse to the faqih and the visit or stay in the mausoleum can treat epilepsy,” “To manage the seizure, putting a key in the hand of the person having a seizure,” and “It is appropriate to put honey, water or medicine in the mouth of the person having a seizure.” (Tables S1–S3).

## 4 DISCUSSION

As far as we know, this is the first study about the understanding and perception of epilepsy among the population of the Souss‐Massa region in southern Morocco. The results of the present study reveal the lack of knowledge and the persistence of myths and confusion concerning epilepsy in the study sample. This statement is consistent with the results of national and international studies. Indeed, a recent survey done by Moroccan teachers revealed a low level of knowledge about epilepsy.^14^ In addition, according to a study conducted in Niger, the level of knowledge about epilepsy is low and tainted with misconceptions and gaps. Only 35.7% of respondents choose doctors, while 38.8% recommend alternative ways of treatment. When asked about the best safety measures to take during crises, about half of the respondents would pray, apply olive oil, run away, or do nothing.^15^ According to another study conducted in Ethiopia, 37.4% of participants believe that epilepsy is God's punishment for sins.^16^

In addition, a study conducted in Sudan raises a low level of knowledge about epilepsy among the population.^17^ Similarly, the one performed in northern Uganda demonstrates little knowledge about epilepsy among adults.^18^ Again, the results of a recent study among Libyan university students in Benghazi show that 57.5% of participants considered epilepsy a psychiatric disorder. Moreover, 37.5% believed in ancient superstitions, such as possession by evil forces, as the underlying cause of the disease. 31.8% recommended traditional remedies to treat it.^19^

Similarly, a study in Saudi Arabia indicates that 20% of participants believed that epilepsy is contagious. Furthermore, 40.1% are convinced that it is due to a spiritual reason.^6^ Likewise, according to a study conducted among the Kuwaiti population, 88.3% indicated that putting an object in the patient's mouth to prevent him from biting his tongue during a seizure is appropriate.^20^

Furthermore, according to the results of a survey conducted among a sample of people with a high level of education in Tehran, 17% indicated that the will of God was the cause.^21^ Yet, according to a recent study conducted in Russia, only 21% of respondents considered epilepsy a neurological disorder, While 34% were afraid to stay near people with epilepsy at the time of the seizure.^22^ Similarly, according to the study conducted in Turkey, participants' knowledge level about epilepsy was low.^23^, ^24^ The results of a study conducted in northeast Thailand state that most participants were unaware of the causes and types of epilepsy. Moreover, more than half of the participants (58%) believed that epilepsy could never be cured.^25^

A study conducted in Greece found that 19% of participants thought epilepsy was a type of mental retardation, 15% thought it was a type of insanity, 91.8% considered epilepsy a brain disorder, and 5.2% a supernatural phenomenon.^26^ Furthermore, a systemic review of studies conducted in developing countries in the Americas, Australia, and Europe has raised inaccurate beliefs among the population regarding the causes, treatment, and prognosis of epilepsy.^27^

Moreover, according to the present study data, negative attitudes toward people living with epilepsy have been detected in a significant part of the sample. Similarly, the outcome of another study conducted among teachers in Morocco revealed the persistence of negative attitudes toward people with epilepsy.^14^ According to several studies, negative attitudes and stigma toward people with epilepsy are still raised.^1^, ^15^, ^22^ This observation was common in several studies in Africa, Asia, and several Arab countries. Again, this finding is raised even among health professionals.^28^, ^29^

In a recent study conducted in Saudi Arabia, half of the respondents admitted that society stigmatizes patients with epilepsy.^6^ Similarly, according to another study, almost half of the sample reported that society discriminates against people with epilepsy.^30^ However, in another study, only 28% were willing to allow a family member to marry someone with epilepsy.^21^ According to the results of a recent study of Libyan university students in Benghazi, 66.6% of participants considered epilepsy to be a barrier to career opportunities, 41% indicated that it was a barrier to practicing sports when 35% did consider it an obstacle to marriage.^19^ Based on a study done in Ethiopia, 51.6% had an unfavorable attitude toward people with epilepsy.^16^ Another scrutiny done among the Kuwaiti populace reported objections to shaking hands with people with epilepsy, working with them, marrying them, and employing them by 16%, 24%, 71%, and 45%, respectively.^20^

Due to stigma, people with epilepsy feel the need to hide their disease for fear of losing their job or being badly treated at work.^24^, ^31^ Furthermore, unemployed people with epilepsy in Iran reported higher levels of perceived stigma than the employed ones.^21^

Furthermore, the analyzed data of the present study shows that good knowledge was significantly associated with sociodemographic factors: age range of more than 54 years and Health professional status. The favorable attitude toward epilepsy correlates to the sociodemographic factors: male gender and Residential urban area. Indeed, the results of a study conducted in Saudi Arabia in 2021 revealed a statistically significant association between a higher level of education and correct beliefs about epilepsy, such as epilepsy is due to a neurological disorder or genetics and that epileptic patients are neither violent nor dangerous and can lead ordinary and successful lives.^6^ Again, the outcome of a study conducted in Kuwait showed that the majority of negative attitudes are significantly associated with a lack of epilepsy awareness. The study concluded that effective and ongoing educational interventions would be needed to improve the understanding of epilepsy and to eliminate social discrimination and misconceptions against epileptic patients.^20^

Negative attitudes and insufficient knowledge among the public in Sharjah, United Arab Emirates, likely contribute to the stigma and discrimination of people with epilepsy in this region. Thus, educational programs are urgently needed to raise awareness and improve public knowledge and attitudes.^30^ In addition, according to a study conducted in southern Ethiopia, lack of acknowledgment, age, and the perception of epilepsy as a punishment from God for sinful activities were variables significantly associated with unfavorable attitudes toward people with epilepsy.^16^ Similarly, another study showed that negative predictors of stigma were age, low level of education, lack of knowledge about epilepsy, and mistaken beliefs about epilepsy (^26^). Overall, the level of understanding of epilepsy was proportional to the education level of the respondents.^22^, ^23^

Again, according to the results of another study, age, gender, low income, and knowledge about epilepsy were significantly associated with negative attitudes toward people with epilepsy.^25^ Similarly, according to a study conducted in the rural areas of Gedeo Zone Southern Ethiopia, unfavorable attitude and age were factors associated with a lack of knowledge of epilepsy.^16^ In another study, misinformation was reported as negatively correlated to positive attitudes toward someone with epilepsy.^32^ Thus, a more positive attitude is related to good knowledge of epilepsy with increasing levels of education. It indicates that negative attitudes are a result of a lack of information. Thus, ensuring enough knowledge about epilepsy will grow positive attitudes in society toward epileptic patients.^33^

## 5 CONCLUSION

The findings of this study suggest a relatively low level of knowledge about epilepsy and a favorable attitude among the sampled population. Good knowledge was significantly associated with sociodemographic factors: age group of more than 54 years and Health professional status. Also, the favorable attitude toward epilepsy notably correlates to the sociodemographic factors: male gender and Residential urban area. The results obtained from this study have the potential to improve awareness and understanding of epilepsy.

## 6 LIMITATIONS

There are several limitations to our analysis. Firstly, our narrow sample study was 385 participants. There was a large number of people who refused to participate in an epilepsy survey. They say that they are afraid to discuss it. This may constitute a bias since these people probably believe in the myths about epilepsy. Second, data collection was limited to areas of the selected regions that are accessible to surveyors. Third, the number of participants in some categories of the sociodemographic variables used in the regression models was small.

## FUNDING INFORMATION

Not applicable.

## CONFLICT OF INTEREST STATEMENT

Both authors have completed The International Committee of Medical Journal Editors (uniform disclosure form) for conflicts of interest. The authors declare that they have no conflict of interest.

## ETHICS APPROVAL STATEMENT AND PATIENT CONSENT STATEMENT

We confirm that we have read the journal's position on issues involved in ethical publication and affirm that this report is consistent with those guidelines. The authors are accountable for all aspects of the work while ensuring the questions related to the accuracy or integrity of any part of the work, and they are appropriately investigated and resolved. Data were collected after obtaining written consent from participants, and all information obtained was kept confidential during all stages of the study. The collected data were used for study purpose only.

## SUBMISSIONS

AT conceived the idea, wrote the proposal, participated in data collection, analysis, and writing up, and reviewed the manuscript. NA participated in data analysis and revised subsequent drafts of the paper. Both authors read and approved this version of the manuscript to be considered for publication.

## Supporting information


Appendix S1.

Appendix S2.

Table S1–S3.

## Data Availability

All raw data included in the manuscript can be accessed from the corresponding author through the email address “aminatani33@gmail.com” with a rational inquiry.
