# Supplementary figures and images for: “The understanding and attitude toward epilepsy in the Souss‐Massa region of Morocco”
Source: Epilepsia Open. 2023 Feb 27;8(2):425–35. doi: 10.1002/epi4.12709 (PMC10235564; doi:10.1002/epi4.12709)

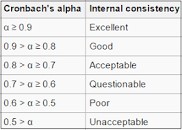

Supplement: Supplementary file 1 — Appendix S1. [file EPI4-8-425-s002.jpg]

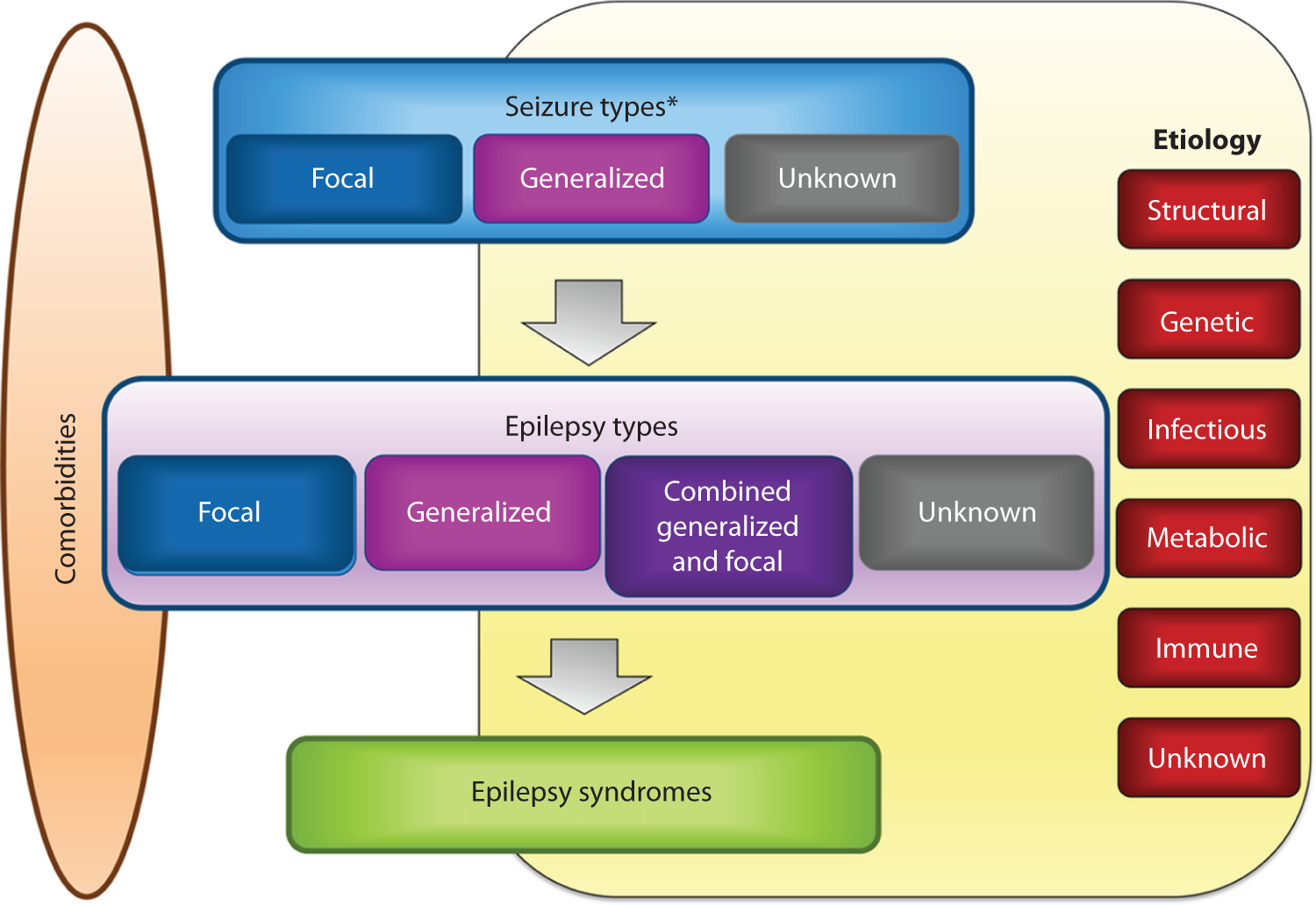

Supplement: Supplementary file 2 — Appendix S2. [file EPI4-8-425-s003.png]
