# Supplementary material for: “The understanding and attitude toward epilepsy in the Souss‐Massa region of Morocco”
Source: Epilepsia Open. 2023 Feb 27;8(2):425–35. doi: 10.1002/epi4.12709 (PMC10235564; doi:10.1002/epi4.12709)
Supplement: Supplementary file 3 — Table S1–S3. [file EPI4-8-425-s001.docx]

Supplement table S1. Outcomes to questions in relation to myths and misunderstandings regarding definitions and causes of epilepsy, differences by sociodemographic variables (n = 385)

| Outcomes to questions in relation to myths and misunderstandings regarding definitions and causes of epilepsy, differences by sociodemographic variables (n = 385) | | | | | | | | | | | | | |
| --- | --- | --- | --- | --- | --- | --- | --- | --- | --- | --- | --- | --- | --- |
|  | | Is epilepsy a psychiatric disorder ? | | | | | | | | Is epilepsy synonymous with madness ? | | | |
| Respondant  characteristic | | Yes | | No | | OR  (95%CI) | | P-value | | Yes | No | OR  (95%CI) | P-value |
| Gender | | | | | | | | | | | | | |
| Male | | 45 | | 115 | | 2.02 (1.167-3.494) | | 0.012 | | 40 | 120 | 2.057 (1.057-9.772) | 0.044 |
| Female | | 30 | | 194 | | reference | |  | | 23 | 202 | Reference |  |
| Age (years) | |  | |  | |  | | 0.389 | |  |  |  | 0.025 |
| 15-34 | | 49 | | 214 | | 0.448(0.142-1.411) | | 0.17 | | 22 | 241 | 0.716(0.254-2.019) | 0.528 |
| 35-54 | | 17 | | 73 | | 0.515(0.165-1.608) | | 0.253 | | 27 | 64 | 2.061(0.738-5.757) | 0.167 |
| More than 54 | | 9 | | 22 | | reference | |  | | 14 | 17 | Reference |  |
| Level of education | |  | |  | |  | | 0.158 | |  |  |  | 0.009 |
| Illiterate | | 9 | | 47 | | 0.425(0.123-1.47) | | 0.177 | | 27 | 30 | 26.437(3.98- 175.595) | 0.001 |
| Primary | | 22 | | 45 | | 1.355(0.557-3.296) | | 0.502 | | 24 | 43 | 15.323(2.506-93.693) | 0.003 |
| Middle/high school | | 19 | | 57 | | 1.234(0.605-2.516) | | 0.564 | | 10 | 66 | 12.241(2.118-70.751) | 0.005 |
| University | | 25 | | 160 | | reference | |  | | 2 | 183 | Reference |  |
| Origin | | | | | | | | | | | | | |
| Urban | | 45 | | 218 | | reference | |  | | 12 | 251 | Reference |  |
| Rural | | 30 | | 91 | | 1.029 (0.492-2.155) | | 0.939 | | 51 | 71 | 4.088(1.71-9.772) | 0.002 |
| Health professional | | | | | | | | | | | | | |
| No | | 71 | | 225 | | 5.687 (1.848-17.5) | | 0.002 | | 61 | 236 | 0.909(0.148-5.602) | 0.918 |
| Yes | | 4 | | 84 | | Reference | |  | | 2 | 86 | Reference |  |
|  | Is epilepsy related to possession by an evil spirit ? | | | | | | | | Is epilepsy related to bewitchment ? | | | | |
| Respondant  characteristic | Yes | | No | | OR (95%CI) | | P-value | | Yes | | No | OR (95%CI) | P-value |
| Gender | | | | | | | | | | | | | |
| male | 72 | | 88 | | 1.862 (1.151-3.013) | | 0,011 | | 63 | | 97 | 1.959 (1.162-3.301) | 0.012 |
| female | 60 | | 165 | | reference | |  | | 45 | | 180 | Reference |  |
| Age (years) |  | |  | |  | | 0.658 | |  | |  |  | 0.461 |
| 15-34 | 78 | | 185 | | 0.981(0.368-2.617) | | 0.970 | | 58 | | 205 | 0.661(0.249-1.753) | 0.405 |
| 35-54 | 35 | | 56 | | 1.292(0.482-3.464) | | 0.610 | | 31 | | 60 |  | 0.006 |
| More than 54 | 19 | | 12 | |  | | reference | | 19 | | 12 | Reference |  |
| Level of education | | | | | | | 0.172 | |  | |  |  | 0.030 |
| Illiterate | 37 | | 20 | | 2.263(0.871-5.877) | | 0.094 | | 33 | | 24 | 3.257(1.187-8.936) | 0.022 |
| Primary | 30 | | 37 | | 0.917(0.408-2.058) | | 0.833 | | 30 | | 37 | 1.996(0.834-4.775) | 0.120 |
| Middle/high school | 27 | | 49 | | 1.173(0.617-2.233) | | 0.626 | | 26 | | 50 | 2.660(1.293-5.469) | 0.008 |
| University | 38+ | | 147 | | reference | |  | | 19 | | 166 | Reference |  |
| Origin | | | | | | | | | | | | | |
| urbain | 63 | | 200 | | reference | |  | | 44 | | 219 | Reference |  |
| rural | 69 | | 53 | | 2.184(1.156-4.127) | | 0.016 | | 64 | | 58 | 2.302(1.184-4.477) | 0.014 |
| Health professional | | | | | | | | | | | | | |
| No | 124 | | 173 | | 4.495(1.910-10.579) | | 0.001 | | 84 | | 193 | 4.411(1.419-13.716) | 0.010 |
| Yes | 8 | | 80 | | Reference | |  | | 4 | | 104 | Reference |  |

OR : Odds Ratio ; CI : Confidence Interval; n : number ; % : percent

Supplement table S2. Outcomes to questions in relation to myths and misunderstandings regarding treatment of epilepsy, differences by sociodemographic variables (n = 385)

| Outcomes to questions in relation to myths and misunderstandings regarding treatment of epilepsy, differences by sociodemographic variables (n = 385) | | | | | | | | |
| --- | --- | --- | --- | --- | --- | --- | --- | --- |
|  | Can traditional practices treat or cure epilepsy ? | | | | Can Quranic Therapy treat or cure epilepsy ? | | | |
| Respondant  characteristic | Yes | No | OR (95%CI) | P-value | Yes | No | OR (95%CI) | P-value |
| Gender | | | | | | | | |
| male | 28 | 132 | 3.460 (1.441-8.308) | 0.005 | 70 | 90 | 1.762(1.093-2.842) | 0.020 |
| female | 9 | 216 | reference |  | 61 | 164 | Reference |  |
| Age (years) |  |  |  | 0.141 |  |  |  | 0.699 |
| 15-34 | 11 | 252 | 0.355(0.104-1.210) | 0.098 | 80 | 183 | 0.896(0.346-2.319) | 0.820 |
| 35-54 | 15 | 76 | 0.839(0.276-2.554) | 0.758 | 33 | 58 | 1.168(0.450-3.028) | 0.750 |
| More than 54 | 11 | 20 | reference |  | 18 | 13 | Reference |  |
| Level of education | | | | 0.622 |  |  |  | 0.101 |
| Illiterate | 15 | 42 | 1.7(0.321-9.008) | 0.533 | 34 | 23 | 3.099(1.203-7.983) | 0.019 |
| Primary | 15 | 52 | 1.564(0.346-7.073) | 0.561 | 30 | 37 | 1.533( 0.694-3.387) | 0.291 |
| Middle/high school | 2 | 74 | 0.535(0.087-3.305) | 0.501 | 31 | 45 | 1.559(0.831-2.922) | 0.166 |
| University | 5 | 180 | reference |  | 36 | 149 | Reference |  |
| Origin | | | | | | | | |
| Urban | 6 | 257 | reference |  | 72 | 191 | Reference |  |
| Rural | 31 | 91 | 5.771(1.669-19.947) | 0.006 | 59 | 63 | 0.934(0.489-1.782) | 0.835 |
| Health professional | | | | | | | | |
| No | 35 | 262 | 1.343(0.222-8.125) | 0.748 | 126 | 171 | 8.192(2.988-22.460) | 0.000 |
| Yes | 2 | 86 | reference |  | 5 | 83 | Reference |  |
|  | Epilepsy can be treated with faqih ? | | | | Epilepsy can be treated by visiting and staying at mausoleums ? | | | |
| Respondant  characteristic | Yes | No | OR (95%CI) | P-value | Yes | No | OR (95%CI) | P-value |
| Gender | | | | | | | | |
| Male | 50 | 110 | 1.932(1.012-3.686) | 0.046 | 44 | 115 | 3.171(1.49-6.752) | 0.003 |
| Female | 31 | 194 | reference |  | 17 | 208 | Reference |  |
| Age (years) |  |  |  | 0.006 |  |  |  | 0.001 |
| 15-34 | 30 | 233 | 0.274(0.094-0.8) | 0.018 | 17 | 245 | 0.175(0.056-0.547) | 0.003 |
| 35-54 | 30 | 61 | 0.804(0.277-2.333) | 0.688 | 26 | 65 | 0,802(0,273-2,35) | 0.687 |
| More than 54 | 21 | 10 | Reference |  | 18 | 13 | Reference |  |
| Level of education | | | | 0.631 |  |  |  | 0.589 |
| Illiterate | 33 | 24 | 1.98(0.612-6.407) | 0.254 | 24 | 33 | 1.972(0.434-8.956) | 0.379 |
| Primary | 28 | 39 | 1.351(0.474-3.849) | 0.573 | 26 | 40 | 2.532(0.66-9.721) | 0.175 |
| Middle/high school | 9 | 67 | 0.98(0.35-2.741) | 0.969 | 7 | 69 | 1.87(0.478-7.315) | 0.368 |
| University | 11 | 174 | Reference |  | 4 | 181 | Reference |  |
| Origin | | | | | | | | |
| Urban | 17 | 246 | Reference |  | 9 | 254 | Reference |  |
| Rural | 64 | 58 | 6.163(2.797-13.58) | 0.000 | 52 | 69 | 6.365 (2.447-16.559) | 0.000 |
| Health professional | | | | | | | | |
| No | 79 | 218 | 5.361(1.106-25.991) | 0.037 | 61 | 235 | 85751204.52 (0.000) | 0.996 |
| Yes | 2 | 86 | Reference |  | 0 | 88 | Reference |  |

OR : Odds Ratio ; CI: Confidence Interval; n : number ; % : percent

Supplement table S3. Outcomes to questions in relation to myths and misunderstandings regarding seizure management of epilepsy, differences by sociodemographic variables (n = 385)

| Outcomes to questions in relation to myths and misunderstandings regarding seizure management of epilepsy, differences by sociodemographic variables (n = 385) | | | | | | | | |
| --- | --- | --- | --- | --- | --- | --- | --- | --- |
|  | During seizure management, do you put water, honey or medication in the person with epilepsy's mouth ? | | | | During seizure management, do you put a key in the person with epilepsy's hand ? | | | |
| Respondant  characteristic | Yes | No | OR (95%CI) | P-value | Yes | No | OR (95%CI) | P-value |
| Gender | | | | | | | | |
| male | 41 | 119 | 1.256(0.695-2.27) | 0.45 | 45 | 115 | 1.385(0.8-2.399) | 0.245 |
| female | 34 | 191 | reference |  | 39 | 186 | Reference |  |
| Age (years) |  |  |  | 0.013 |  |  |  | 0.444 |
| 15-34 | 32 | 231 | 0.278(0.103-0.746) | 0.011 | 44 | 219 | 0.599(0.23-1.557) | 0.293 |
| 35-54 | 25 | 66 | 0.65(0.249-1.695) | 0.378 | 24 | 67 | 0.861(0.334-2.218) | 0.756 |
| More than 54 | 18 | 13 | reference |  | 16 | 15 | Reference |  |
| Level of education | | | | 0.046 |  |  |  | 0.016 |
| Illiterate | 26 | 31 | 4.184(1.307-13.388) | 0.016 | 27 | 30 | 5.193(1.798-14.998) | 0.002 |
| Primary | 25 | 42 | 4.058(1.458-11.294) | 0.007 | 25 | 42 | 3.67(1.457-9.246) | 0.006 |
| Middle/high school | 15 | 61 | 2.695(1.08-6.725) | 0.034 | 18 | 58 | 2.282(1.03-5.058) | 0.042 |
| University | 9 | 176 | reference |  | 14 | 171 | Reference |  |
| Origin | | | | | | | | |
| urban | 30 | 233 | reference |  | 39 | 224 | Reference |  |
| rural | 45 | 77 | 1.126(0.531-2.387) | 0.758 | 45 | 77 | 0.907(0.444-1.854) | 0.789 |
| Health professional | | | | | | | | |
| No | 74 | 223 | 11.556(1.429-93.432) | 0.022 | 82 | 215 | 7.175(1.573-32.74) | 0.011 |
| Yes | 1 | 87 | reference |  | 2 | 86 | Reference |  |

OR : Odds Ratio ; CI: Confidence Interval; n : number ; % : percent
